# Improved YOLOv11n-seg for impurity detection in mechanically harvested sugarcane

**DOI:** 10.3389/fpls.2026.1745861

**Published:** 2026-02-18

**Authors:** Fengguang He, Sili Zhou, Pinlan Chen, Ganran Deng, Shaobo Feng, Guojie Li, Zhende Cui, Shuang Zheng, Ling Li, Bin Yan, Shuangmei Qin, Xilin Wang, Ye Dai, Zehua Liu

**Affiliations:** 1 Agricultural Machinery Research Institute, Chinese Academy of Tropical Agricultural Sciences, Zhanjiang, Guangdong, China; 2 Key Laboratory of Tropical Agricultural Machinery, Ministry of Agriculture and Rural Affairs, Zhanjiang, Guangdong, China; 3 Guangxi Research Institute of Metrology & Test, Nanning, Guangxi, China

**Keywords:** sugarcane, impurity detection, instance segmentation, YOLOv11, deep learning, lightweight

## Abstract

The content of impurities in mechanically harvested sugarcane is a critical factor for evaluating harvest quality and determining market price. To enable intelligent detection of impurities in mechanically harvested sugarcane, this study proposes an impurity detection method based on an improved YOLOv11n-seg model. The method integrates four enhancement modules into the original YOLOv11n-seg architecture. Firstly, a lightweight C2_Ghost module is introduced into the high-channel feature extraction stages of both the backbone and neck, thereby reducing computational complexity and feature redundancy. Subsequently, a C2_FSAS module is designed to perform frequency-domain relationship modelling, enhancing long-range semantic dependency representation. An Efficient Channel Attention (ECA) mechanism is then applied to deep high-level semantic features to adaptively reweight salient feature channels. Finally, the traditional fixed interpolation-based upsampling operation is replaced with a dynamic DySample upsampling strategy to recover fine-grained edge features. Experimental results indicate that Improved YOLOv11n-seg achieves segmentation performance of 97.0%, 98.1%, 99.2%, and 82.9% in terms of P, R, mAP_0.5_, and mAP_0.5:0.95_, respectively. Compared with the original YOLOv11n-seg, the proposed model achieves a 1.8% improvement in mAP0.5:0.95, a 10.2% reduction in parameter count, and maintains a real-time inference speed of 34.8 FPS on the Jetson Xavier NX under TensorRT acceleration. Ablation studies validate the effectiveness of the four-module synergistic design, with C2_FSAS and DySample contributing most significantly to the improvement in mAP. Moreover, the model exhibits enhanced edge delineation accuracy and inter-class discrimination capability. In summary, the Improved YOLOv11n-seg achieves a favourable balance between segmentation accuracy and real-time performance, enabling precise segmentation of sugarcane segments and diverse impurity types. The proposed method provides reliable technical support for intelligent impurity rate detection in mechanically harvested sugarcane and practical deployment on edge computing platforms.

## 1 Introduction

As one of the world’s most important sugar crops, sugarcane production is essential for safeguarding sugar security and supporting economic development (Liu et al., 2020). In 2023, China’s sugarcane production exceeded 100 million tons (Food and Agriculture Organization of the United Nations, 2025). The State Council Document [2018] No. 42 specifies that China’s sugarcane harvesting mechanization rate must reach 30% by 2025 (Liu et al., 2024; Zhou et al., 2024). However, mechanized harvesting inevitably introduces various impurities (Que et al., 2024)—including sugarcane tops, leaves, roots, and soil. Mechanized sugarcane harvesting can reduce soil impurity intake through optimized cutting height adjustment (Shi et al., 2024) and by improving the conveying and cutting systems to remove sugarcane leaves and soil debris (Afsharnia et al., 2025; Zhou et al., 2025a). The cut sugarcane and associated impurities are conveyed into the impurity-removal fan channel. By leveraging the differences in suspension velocities between sugarcane segments and impurities, lighter components such as sugarcane tops and leaves are removed (Zhou et al., 2025b). However, excessively low fan speeds lead to impurity retention, whereas excessively high speeds may cause unintended removal of sugarcane segments. Therefore, the optimization of variable-speed fan control strategies can effectively reduce impurity content (Wu et al., 2024). Although mechanized sugarcane harvesting currently removes over 90% of impurities, residual impurities remain, and impurity levels cannot be monitored during post-fan collection. Due to limitations in current sugar production equipment and processing technologies in China, sugar mills require raw sugarcane impurity rates to be below 5% (Li et al., 2024; Zhou et al., 2025a; Zhou et al., 2025c). Impurities significantly reduce the purity of raw sugarcane, directly impacting sugar yield and quality, accelerating equipment wear, increasing energy consumption, and raising production costs (De Mello et al., 2022). As a result, impurity content has become a critical metric for assessing raw material quality, managing production costs, and determining purchase prices in sugar processing. Raw sugarcane undergoes secondary impurity removal procedures—including unloading, feeding, impurity separation, and discharge—at sugar mills. However, these procedures lack the capability for automatic detection of impurity content. Instead, traditional approaches rely on manual sampling and weighing, with impurity levels estimated from randomly selected small-scale samples. These methods are associated with high subjectivity, low efficiency, and considerable variability (Li et al., 2023; Zhou et al., 2024). Therefore, the development of rapid and objective devices for measuring sugarcane impurity levels is urgently required in the sugar industry, and intelligent impurity detection technologies are crucial for advancing such equipment.

In recent years, deep learning has achieved remarkable advancements, and scholars worldwide have conducted extensive research on its application in impurity detection (Ding et al., 2025a). Huang and Liang (2022) integrated a feed forward convolutional attention mechanism, spatial pyramid pooling, and depth wise separable convolutions into a lightweight YOLOv5-based tea impurity detection model, achieving a multi-category mAP of 96.05%. Wang et al (2024) proposed an improved YOLOv5 model for cotton surface impurity detection, incorporating MCA to enhance feature extraction for impurity targets, resulting in a mAP of 92.5%. Yu et al (2023) replaced convolutional blocks with a Transformer-Encoder module to improve global feature representation in walnut kernel impurity detection, while integrating GhostNet reduced detection time by 10.4% and increased mAP to 88.9%. Ding et al (2025b) improved detection performance and model lightweighting by modifying the loss function and employing lightweight convolutions along with model pruning, yielding a 2.6% improvement in mAP and a 37% reduction in inference time. However, bounding-box-based object detection methods are limited in accurately delineating object boundaries for both primary targets and impurities, resulting in suboptimal precision in impurity content estimation.

Image-based instance segmentation methods can identify objects of different categories at the pixel level, enabling precise localization of primary objects and impurity regions. Chen et al., (2023) developed a U-Net-based method for impurity rate detection in machine-harvested wheat. Zhao et al. (2024) enhanced the Mask R-CNN model by incorporating a CA attention mechanism and fully connected layers to detect impurities such as dried leaves, senescent vegetable leaves, and paper fragments in leafy vegetables, achieving a mAP of 98.55%, although the detection speed remained suboptimal. Pan et al. (2025) proposed a potato impurity detection model based on PLP-net, which incorporates an ECA mechanism to enhance feature extraction, achieving an mAP of 96.0%. Qi et al. (2024) introduced a DeepLab-EDA segmentation model for assessing wheat crushing rate and impurity rate, yielding an accuracy of 95.97%. Li et al. (2023) developed the MDSC-DeepLabv3+ model by integrating MobileNetv2, ASPP, depth wise separable convolutions, and CA attention mechanisms for the segmentation and detection of sugarcane impurities including sugarcane segments, tops, and leaves, achieving a mAP of 97.55%. These studies demonstrate that instance segmentation-based approaches enable pixel-level precision analysis, providing a solid technical foundation for subsequent high-precision impurity quantification.

Although mechanical screening and air separation methods can effectively remove most impurities during harvesting, these approaches primarily focus on physical separation processes rather than the perception of impurity composition information. Compared with traditional image processing methods, YOLO-based segmentation frameworks enable real-time, end-to-end detection, classification, and pixel-level segmentation of sugarcane impurities. This approach provides rapid, non-contact, and objective visual perception for assessing impurity levels in mechanically harvested sugarcane. Consequently, this study focuses on impurity rate detection during post-harvest transportation to sugar mills. This study proposes an Improved YOLOv11n-seg (Khanam and Hussain, 2024) method targeting three key biological impurities—leaves, tops, and roots. The model integrates the Ghost module (Han et al., 2020), ECA mechanism (Wang et al., 2020), efficient frequency domain-based self-attention solver (FSAS) (Kong et al., 2023), and DySample upsample module (Liu et al., 2023) to enhance representational capability. This enhancement yields a more robust sugarcane impurity segmentation model, offering technical support for impurity-rate detection equipment in sugarcane processing.

## 2 Materials and methods

### 2.1 Image acquisition

**Figure 1:**
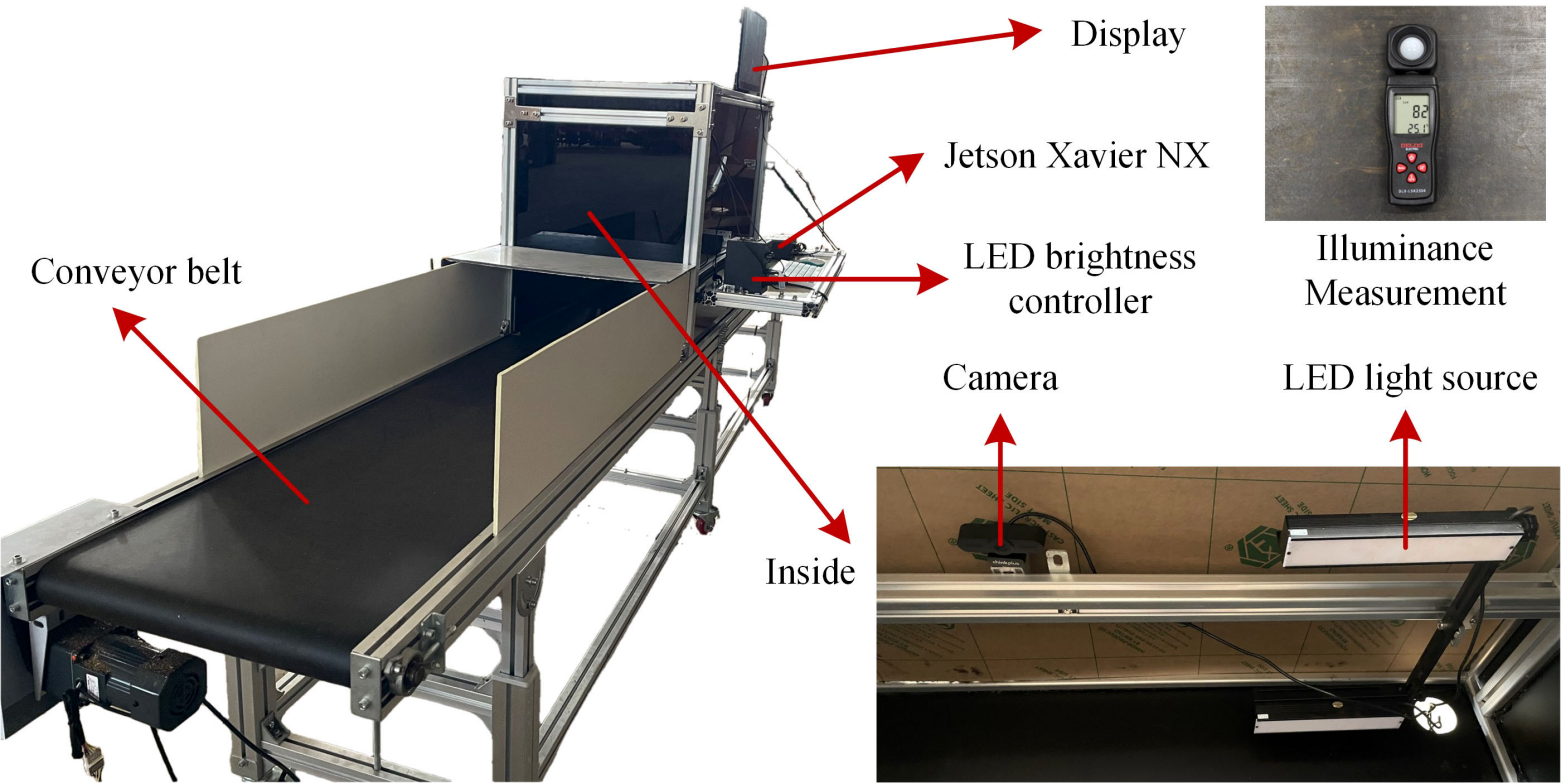
Image acquisition device.

The sugarcane impurity image data used in this study were acquired in July 2025 at the Experimental Base of the Agricultural Machinery Research Institute, Chinese Academy of Tropical Agricultural Sciences in Zhanjiang, Guangdong Province (21.17°N, 110.27°E). **Figure 1** shows the image acquisition device. The camera is equipped with a Lenovo WL24A autofocus lens, offering a maximum resolution of 2592×1944 and a maximum image transmission rate of 30 FPS. It is positioned approximately 45 cm above the conveyor belt and oriented vertically downward. Two LED light sources were arranged on both sides of the camera to ensure consistent illumination. During image acquisition, sugarcane moves along the conveyor belt at a constant speed of approximately 17 cm/s. Simultaneously, the camera captures images at a rate of one frame per second using a Python-based acquisition program. The sugarcane variety used was “Yuetang 94-128” and the captured samples consisted of small segments, reflecting the typical condition of sugarcane following mechanical harvesting.

### 2.2 Preparation of sugarcane impurity dataset

**Figure 2:**
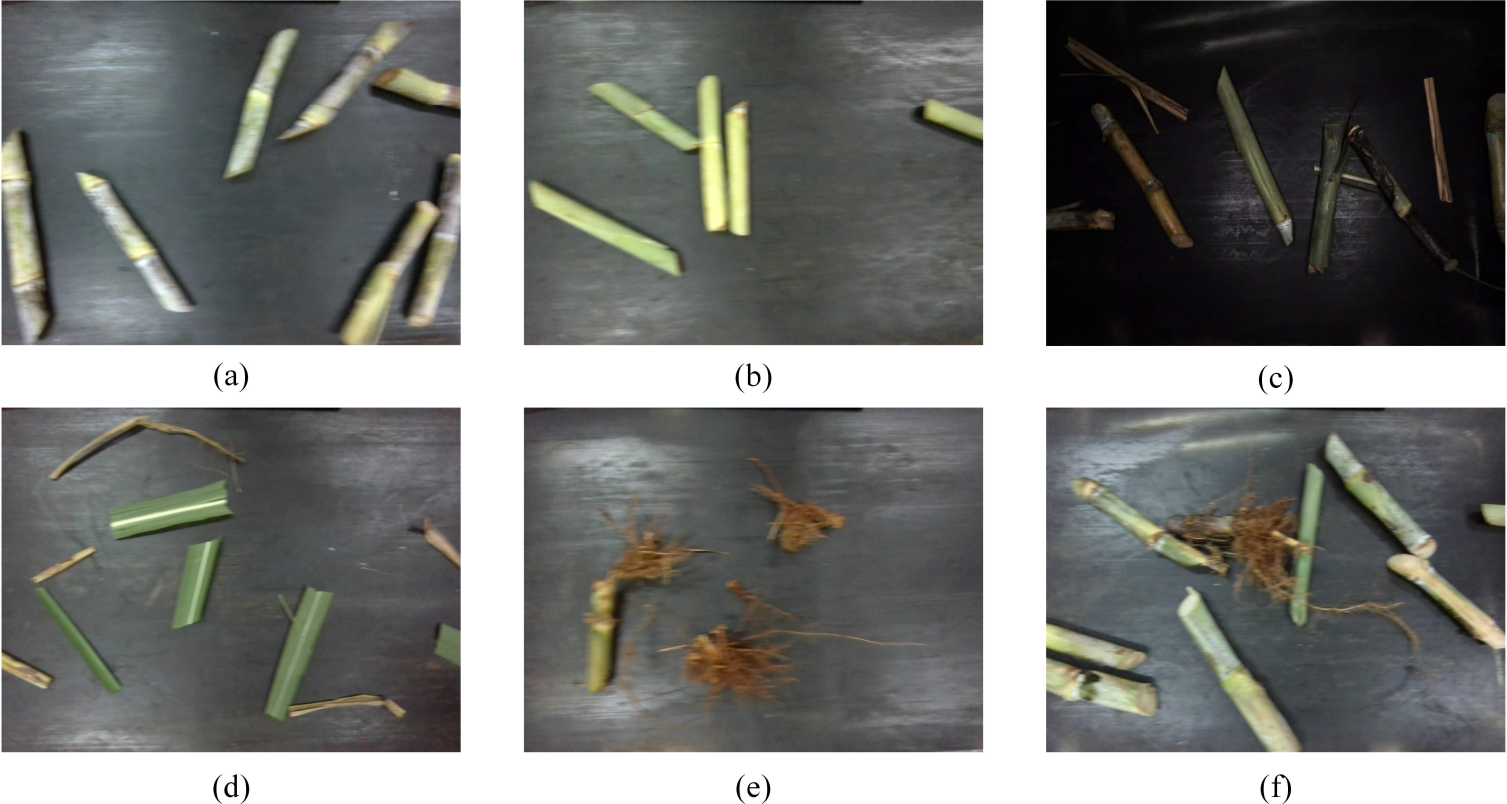
Categories within the dataset. **(a)** cane_segment. **(b)** cane_top. **(c)** mixed_low-light. **(d)** cane_leaves. **(e)** cane_root. **(f)** mixed_well-lit.

**Figure 3:**
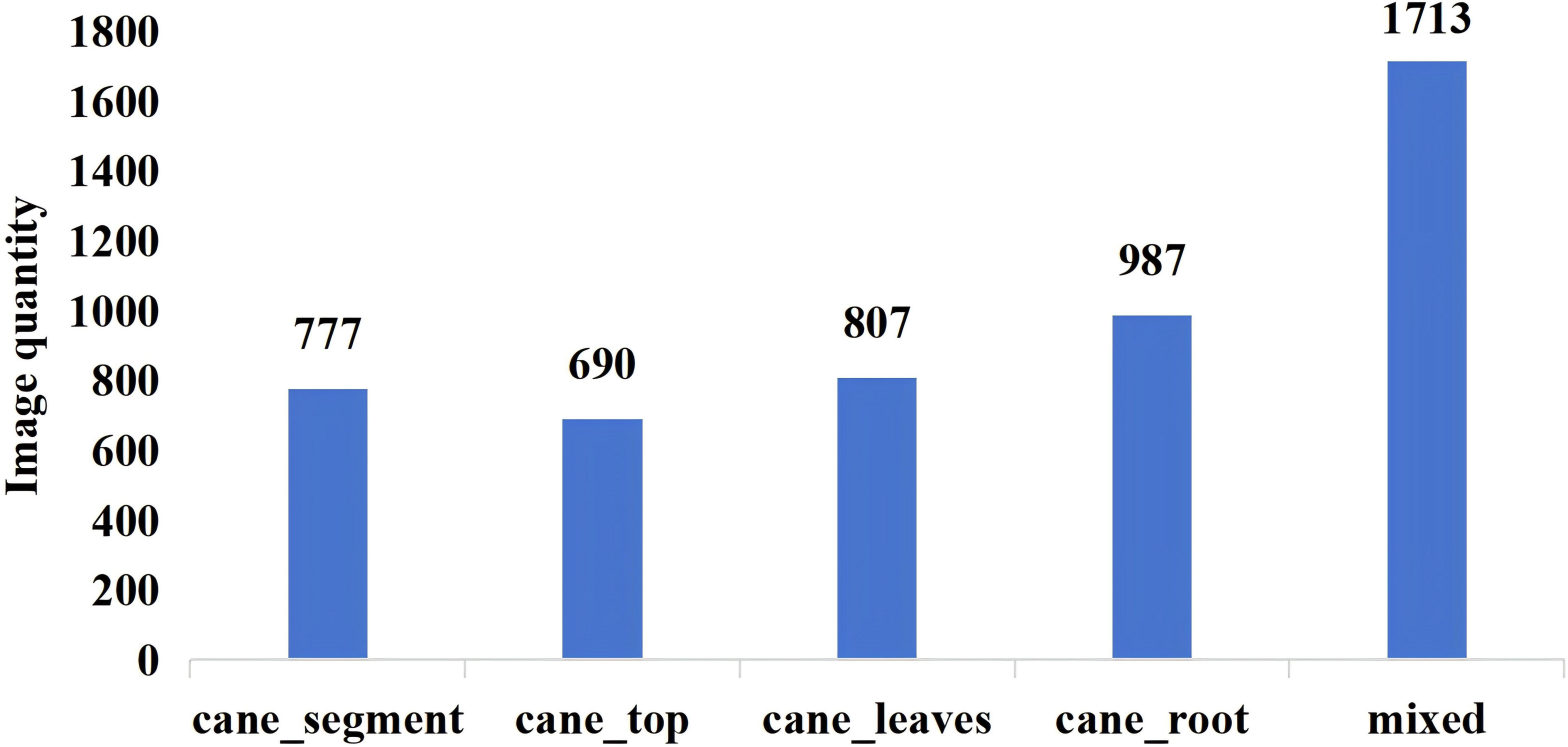
Composition of sugarcane impurity dataset.

Soil impurities can be substantially reduced by adjusting the height between the harvesting device and the ridge surface or through mechanical vibration-assisted cleaning methods (Xie et al., 2018; Martins and Ruiz, 2020). However, impurities such as sugarcane, tops, leaves, and roots cannot be effectively removed during harvesting. Therefore, this study selected sugarcane tops, leaves, and roots as the primary targets for impurity detection. The collected images were manually annotated using X-AnyLabeling software and categorized into four types: sugarcane segments, sugarcane tops, sugarcane leaves, and sugarcane roots, with the corresponding label names cane_segment, cane_top, cane_leaves, and cane_root, and the annotations were in an instance segmentation format. The annotated images cover pure sugarcane segments, pure sugarcane tops, pure sugarcane leaves, pure sugarcane roots, and mixed-category scenarios under varying brightness conditions, as shown in **Figure 2**. Illuminance levels were measured using a DELIXI DLK-LSK2304 illuminance meter under low-light (23 Lux) and well-lit (82 Lux) conditions. In total, 4,974 images were annotated. The aspect ratio of all images was uniformly preserved while resizing the longer side to 640 pixels. **Figure 3** details the dataset composition across all scenarios. Finally, the JSON files produced during annotation were converted into YOLO format text files. To ensure stable model training and improve generalization, the dataset was divided into training, validation, and test sets at a ratio of 8:1:1.

### 2.3 Design of the sugarcane impurity detection model

The YOLOv11-seg backbone network extracts low-level edge and texture information along with high-level semantic features from input images through hierarchical convolutions and multi-scale feature extraction. The neck structure then performs multi-scale feature fusion and enhancement. Finally, the anchor-free detection head jointly outputs bounding boxes, classification confidence, and pixel-level segmentation masks, while non-maximum suppression (NMS) removes redundant predictions. Compared with other YOLO variants and segmentation models such as U-Net (Ronneberger et al., 2015), YOLOv11n-seg offers a better trade-off between detection speed and segmentation accuracy. Furthermore, unlike two-stage instance segmentation models such as Mask R-CNN (He et al., 2017), YOLOv11n-seg utilizes a single-stage end-to-end design that maintains high accuracy and robust real-time performance, making it particularly suitable for deployment on low-power edge devices to meet sugarcane impurity detection requirements.

To address the issue of feature confusion among sugarcane segments, tops, and leaves, as well as the blurry boundaries in sugarcane root segmentation, this study introduces targeted improvements to the YOLOv11n-seg architecture. First, a lightweight Ghost Block-based C2_Ghost module, combining the Ghost module with the C3K2 module from YOLOv11, is applied to the high-channel regions of both the backbone and neck networks, significantly reducing redundant computation while maintaining expressive feature representation. In deeper layers of the backbone, spatial resolution decreases whereas channel dimensions increase, enabling the final C2_Ghost output to aggregate rich high-level semantic information. However, excessive channels may lead to redundancy and imbalance, potentially weakening informative features and retaining irrelevant ones. Therefore, the ECA mechanism is introduced between the C2_Ghost and SPPE modules to enhance channel-wise weighting and improve attention to critical regions.

**Figure 4:**
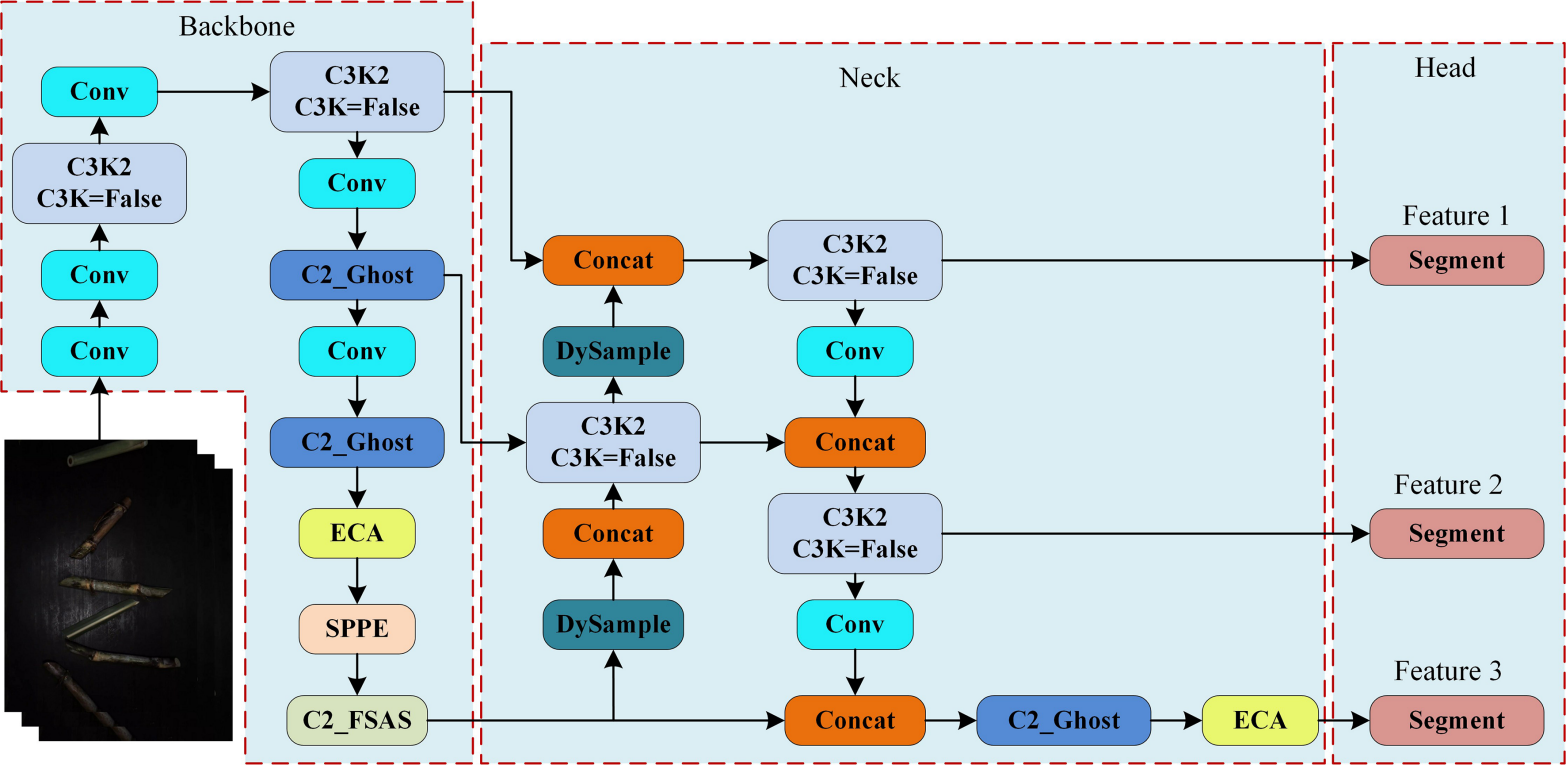
Improved YOLOv11n-seg network structure. (Conv, C3K2, SPPE, and Segment are modules from the original YOLOv11n-seg, where Conv uses SiLU activation by default, and Concat refers feature channel concatenation.).

Next, the PSA module in the original C2PSA structure is replaced with a frequency-domain self-attention solver (FSAS) to construct the C2_FSAS semantic feature extraction module. By transforming feature maps into the frequency domain for global relationship modeling, this module effectively captures long-range dependencies and distinguishes categories with similar shape or color characteristics, further improving impurity recognition accuracy. In the neck network, the traditional UpSample module is replaced with the lightweight and dynamically adjustable DySample module, which adaptively adjusts interpolation weights to restore spatial resolution and reduce edge-blurring effects, thereby enhancing boundary segmentation precision for sugarcane segments and impurities. Additionally, ECA is integrated between the final C3K2 and Segment modules in the neck to emphasize salient channels and suppress redundant ones, improving classification performance for multiple impurity categories. The architecture of the Improved YOLOv11n-seg is illustrated in **Figure 4**.

#### 2.3.1 C2_ghost module

**Figure 5:**
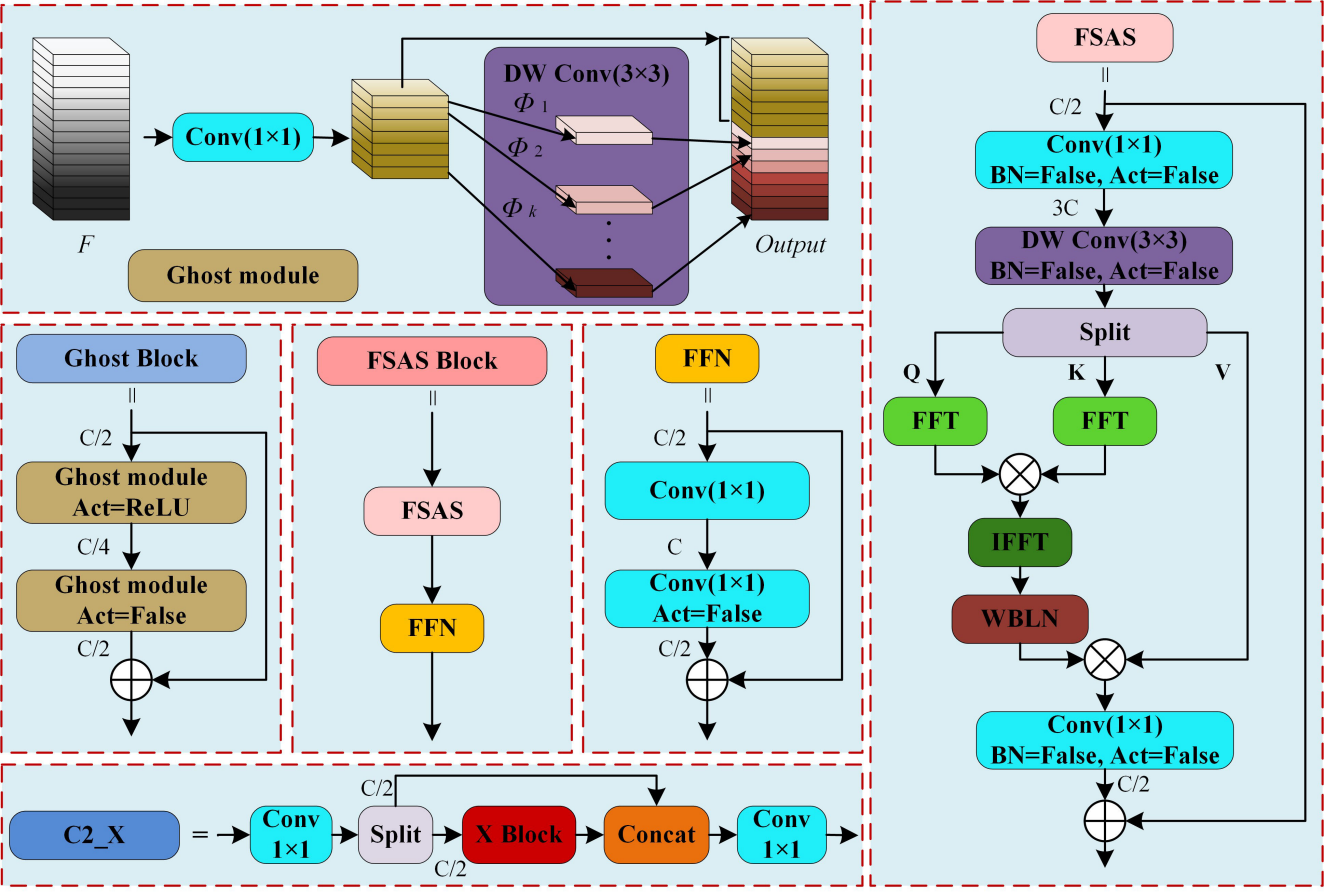
C2_X module structure. (The X Block in the figure refers either a Ghost Block or an FSAS Block, which correspond to the C2_Ghost and C2_FSAS modules respectively. Split refers to average channel partitioning, where C refers the number of channels; DW Conv refers to depth wise convolution; Φ refers low-cost linear operations; FFT and IFFT denote Fast Fourier Transform and Inverse Fast Fourier Transform; WBLN refers With Bias Layer Norm; BN refers to Batch Norm; and Act refers the activation function.).

Convolutional operations may produce redundant feature maps during feature extraction, and these redundant representations can be generated via computationally efficient linear transformations (Han et al., 2020). In the YOLOv11n-seg network, the C3K module used within the C3K2 block is a composite structure consisting of multiple convolutional layers. As the number of feature channels increases, these operations tend to generate a greater number of redundant feature representations, introducing unnecessary computational overhead. To address this issue, the Ghost module efficiently derives redundant feature representations from primary intrinsic features through low-cost linear transformations. Specifically, the Ghost module first employs a standard 1×1 convolution to produce representative intrinsic feature maps. Subsequently, it enhances these intrinsic features by generating additional feature maps through a 3×3 depth wise convolution, which serves as a computationally inexpensive linear operation. Finally, concatenation is applied to maintain consistent input-output channel dimensions, enabling the expression of feature representations equivalent to those from standard convolutions but with significantly reduced computational cost. As shown in the Ghost module section of **Figure 5**, the process can be mathematically formulated as Equation 1.

(1) $$Output=Concat(Conv_{1\times 1}(F),\;DW\;Conv_{3\times 3}(Conv_{1\times 1}(F)))$$

Where *F* refers the input feature map and *Output* refers the output feature map. To achieve high real-time performance and lightweight inference in sugarcane impurity detection, this study replaces the original C3K structure in the C3K2 module with a Ghost Block constructed using Ghost modules, thereby forming the lightweight C2_Ghost feature extraction module. As illustrated in the C2_X structure in **Figure 5**, C2_Ghost sequentially processes feature information via a 1×1 convolution, channel splitting (Split), Ghost Block transformation, channel concatenation (Concat), and a 1×1 convolution. This design retains strong feature representation capability while substantially reducing the number of parameters and computational overhead.

#### 2.3.2 C2_FSAS module

The sugarcane impurity detection task, when conducted in a conveyor belt format, suffers from motion blur. Moreover, sugarcane segments, tops, and leaves share highly similar color, shape, and texture characteristics, making feature discrimination challenging. To enhance the extraction of high-level semantic information and improve category differentiation, the FSAS mechanism is integrated into the C2PSA architecture of YOLOv11n-seg, forming a more efficient feature extraction module that performs global dependency modelling in the frequency domain. Specifically, FSAS utilizes the FFT to convert input feature maps from the spatial domain to the frequency domain, where low-frequency components represent smooth background structures and global contours, whereas high-frequency components preserve fine-grained structural details such as edges and textures. By performing frequency-domain filtering, FSAS effectively captures long-range pixel dependencies with low computational complexity. Subsequently, the refined frequency-domain features are converted back to the spatial domain using the IFFT. As illustrated in the FSAS section of **Figure 5**, this procedure is mathematically formulated in Equations 2, 3.

(2) $$Q,\;K,\;V=Split(DW\;Conv_{3\times 3}(Conv_{1\times 1}(F)))$$

(3) $$Output=F+Conv_{1\times 1}(WBLN(IFFT(FFT(Q)⊗FFT(K)))⊗V)$$

According to the convolution theorem, the convolution or correlation of two features in the spatial domain corresponds to their element-wise product in the frequency domain (Kong et al., 2023). FSAS therefore extracts discriminative features through element-wise multiplication in the frequency domain, enhancing or suppressing specific frequency characteristics of sugarcane target objects. This mitigates detail loss resulting from target motion and yields more accurate category boundary representations. For impurities whose color and texture closely resemble those of sugarcane segments, their spectral distributions still present fine-grained distinctions in the frequency domain. Accordingly, FSAS can more effectively detect inter-class variations in sugarcane impurity detection. Following FSAS, a Feed-Forward Network (FFN) module is introduced to perform both non-linear transformations and inter-channel feature fusion, thus enhancing the model’s representational capacity and classification ability and ultimately forming the FSAS Block. As illustrated in **Figure 5** (C2_X), C2_FSAS processes features sequentially via 1×1 Conv, channel splitting (Split), the FSAS Block, channel concatenation (Concat), and a final 1×1 Conv.

#### 2.3.3 ECA module

**Figure 6:**
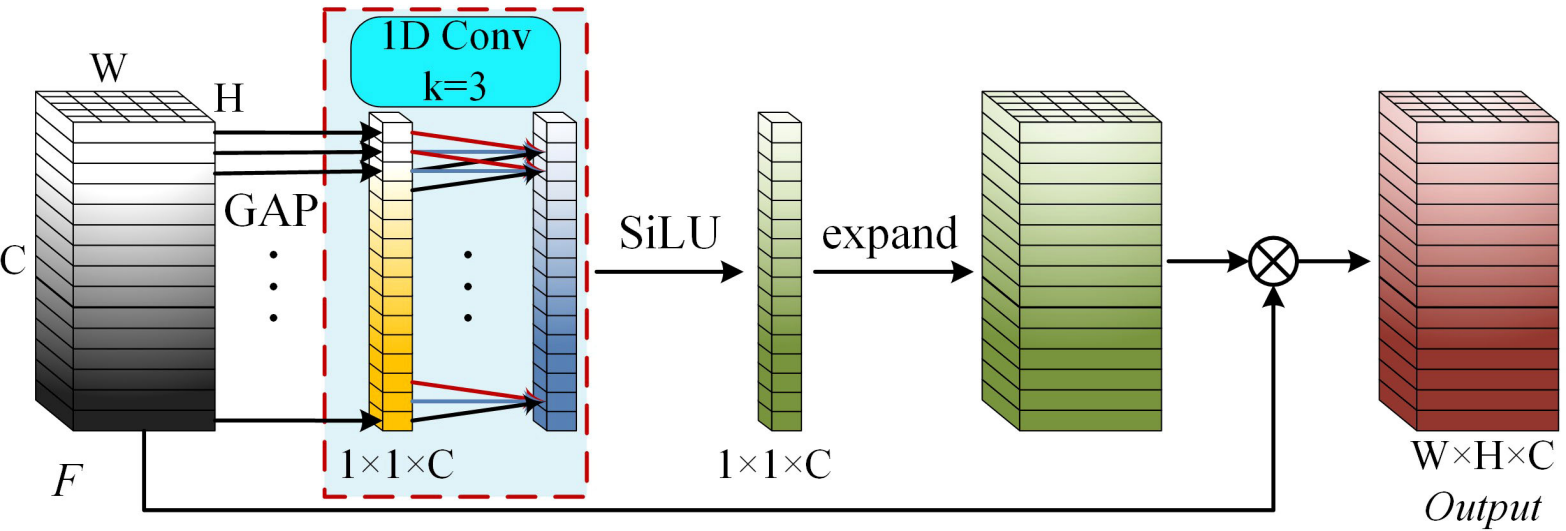
ECA module. (GAP refers to Global Average Pooling; 1D Conv denotes one-dimensional convolution; and expand refers to dimension expansion.).

In the sugarcane impurity detection task, image data include background regions and multiple impurity categories. The contribution of each feature channel to identifying sugarcane segments, leaves, tops and roots differs significantly. With progressive decreases in spatial resolution, the number of feature channels increases substantially, causing deeper layers to aggregate richer semantic information while also introducing substantial feature redundancy. As a result, the discriminative channels associated with specific impurity categories may be suppressed by irrelevant or noisy channels, ultimately degrading detection accuracy. Compared with the SE attention mechanism, ECA eliminates the negative influence of dimensionality reduction on channel attention and achieves higher computational efficiency (Wang et al., 2020). Therefore, this study introduces the ECA mechanism after the C2_Ghost module, which possesses the lowest spatial resolution and the highest channel dimensionality, to reduce the influence of redundant and noisy channels. As illustrated in **Figure 6**, the ECA mechanism is mathematically formulated by Equations 4.

(4) $$Output=F⊗SiLU(1D\;Conv_{1\times 1}(GAP(F)))$$

The ECA mechanism first computes channel-wise average statistics of the input feature map using Global Average Pooling (GAP). A 1D convolution with a kernel size of 3 is then applied to the resulting 1×1×C descriptor to generate channel attention weights with minimal computational overhead. The obtained weights are subsequently broadcast to match the spatial dimensions of the input feature map. Finally, the recalibrated weights are multiplied with the original feature map to enable lightweight local cross-channel interaction.

#### 2.3.4 DySample module

**Figure 7:**
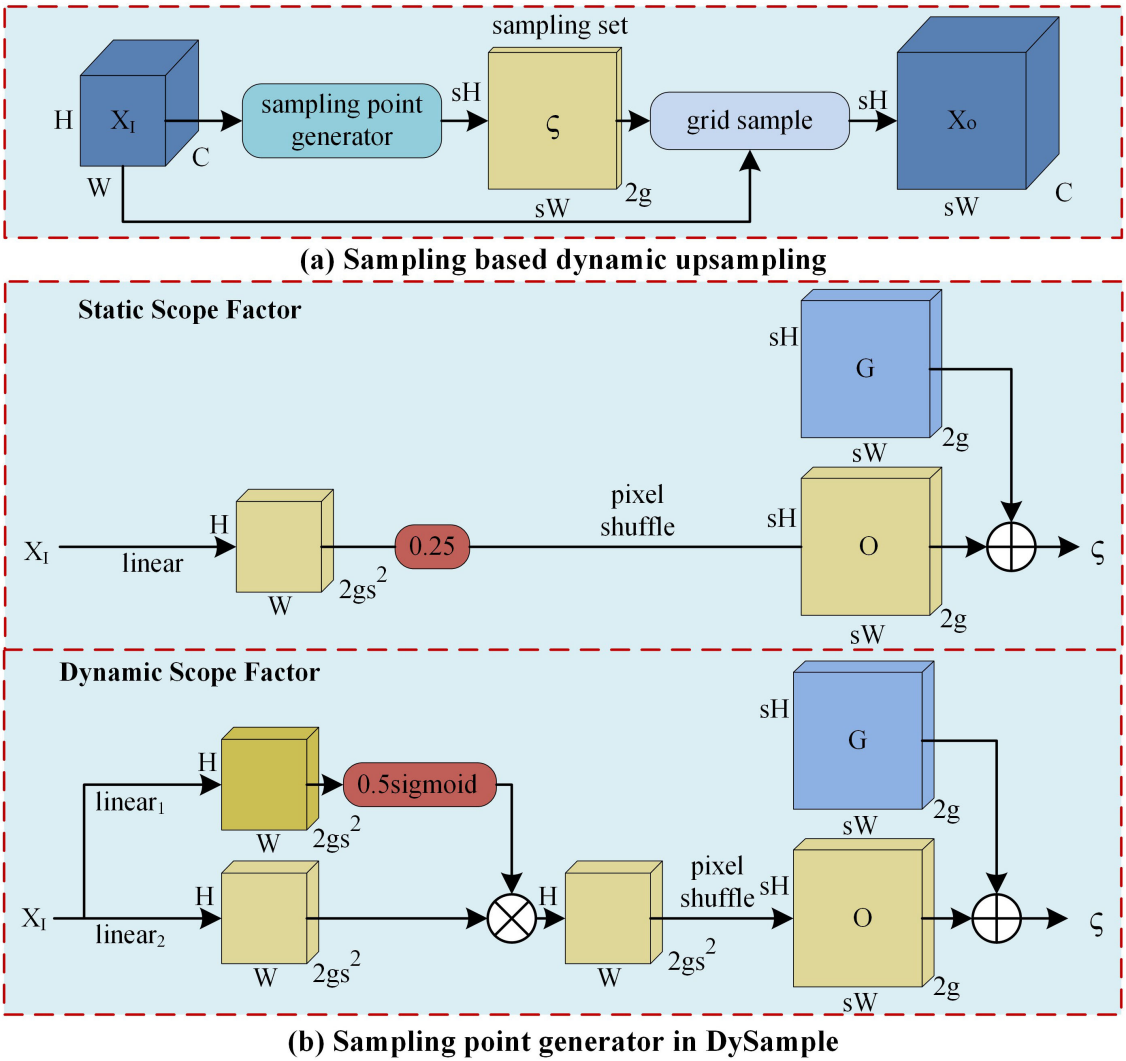
DySample module. (X_I_, X_o_, O, and G denote the input features, upsample features, offset, and original sampling grid, respectively. g refers the grouping number, s refers the upsample ratio, and ς refers to the sampling set.).

upsample is essential for reconstructing accurate pixel-level masks from low-resolution, high-level semantic feature maps. However, impurity boundaries in sugarcane commonly exhibit irregular shapes, and the contours of sugarcane segments and tops are highly similar. Traditional interpolation-based upsample uses fixed interpolation kernels, which can easily result in feature loss and blurred boundaries when processing complex shapes. This reduces localization and segmentation accuracy between sugarcane segments and impurities. To overcome these limitations, the DySample module is incorporated into the neck network to replace the original UpSample operator. DySample utilizes learnable dynamic weights to adaptively refine sampling positions and interpolation weights based on local feature content. This enables differentiated upsample for spatially diverse regions, allowing better preservation of structural details during high-resolution feature reconstruction. Furthermore, DySample is lightweight and does not require custom CUDA implementations, achieving excellent efficiency in terms of parameter count, FLOPs, and inference latency. As illustrated in **Figure 7**, DySample generates the upsample feature X_o_ by feeding the input feature X_I_ and sampling set ς, obtained from the sampling point generator, into the grid_sample function, as formulated in Equation 5.

(5) $$X_{o}=grid\_sample(X_{I},\varsigma )$$

The input features are processed through a linear layer to produce a 2gs^2^×H×W offset, which is subsequently converted via pixel shuffle into a 2g×sH×sW offset O. The static and dynamic offsets are mathematically formulated in Equations 6 and Equations 7, respectively. The sampling set ς is then defined as the sum of offset O and the original sampling grid G, as formulated in **Equation 8.**

(6) $$O=pixel\;shuffle(0.25\times linear(X_{I}))$$

(7) $$O=pixel\;shuffle(0.5\times sigmoid(linear_{1}(X_{I}))+linear_{2}(X_{I}))$$

(8) ς=G+O

### 2.4 Model training configuration

The hardware configuration for model training in this study consisted of an Intel^®^ Xeon^®^ Gold 6256 CPU @ 3.60GHz and an NVIDIA RTX A6000 GPU with 48 GB. The software environment was based on Ubuntu 20.04, using CUDA 11.8 and cuDNN 8.6.0 to accelerate deep learning computations. A dedicated Anaconda virtual environment was created for model training, with key dependencies including PyTorch 2.0.1 and OpenCV 4.10, and Python 3.10 as the programming language. During training, the number of epochs was set to 200, the batch size was 32, and the initial learning rate was 0.01. The AdamW optimizer was employed, and 4 data loader workers were used.

### 2.5 Evaluation metrics

Model testing was conducted on a Jetson Xavier NX edge device running JetPack 5.1, configured with TensorRT 8.5.2.2, CUDA 11.4, and cuDNN 8.6.0, and equipped with 16 GB of GPU memory. To objectively evaluate the performance of the Improved YOLOv11n-seg model, the following evaluation metrics were utilized: Precision (P), Recall (R), Mean Average Precision (mAP), parameter count, and inference speed (FPS). P measures the proportion of correctly predicted positive samples, indicating the model’s ability to control false positives. R measures the proportion of actual positive samples correctly identified, reflecting its effectiveness in minimizing false negatives. mAP provides a comprehensive assessment of detection and segmentation performance based on the mean accuracy over all categories. The number of parameters reflects model complexity, where fewer parameters indicate greater suitability for deployment on resource-limited devices. FPS measures the number of frames processed per second, with higher FPS indicating faster inference and improved efficiency in real-time applications.

## 3 Results and analysis

### 3.1 Comparison of different object detection models

This study utilized five YOLO-based instance segmentation models, all trained using an identical dataset. Each model was trained for 200 epochs, and the best-performing weights were selected as the final model parameters. The performance comparison of all models on the test set is presented in **Table 1**.

**Table 1:** Performance comparison of different YOLO-based instance segmentation models on the test set.

Model	P	R	mAP_0.5_	mAP_0.5:0.95_	Parameters(×10^5^)	Speed(FPS)
YOLOv5n-seg	96.3	95.7	98.4	80.2	27.6	19.6
YOLOv8n-seg	96.1	96.2	98.5	81.8	32.6	19.4
YOLOv9t-seg	96.6	95.7	98.6	81.1	**23.8**	10.0
YOLOv10n-seg	96.6	96.3	98.7	81.2	25.2	**20.0**
YOLOv11n-seg	95.6	96.3	98.3	81.1	28.4	18.0
YOLOv12n-seg	95.4	96.8	98.3	81.1	28.1	14.2
Improved YOLOv11n-seg	**97.0**	**98.1**	**99.2**	**82.9**	25.5	18.0

Bold text indicates optimal values.

The results demonstrate that the proposed Improved YOLOv11n-seg model exhibits superior performance across key metrics, including P, R, and mAP, confirming the effectiveness and soundness of the model enhancement strategy. Regarding detection accuracy, Improved YOLOv11n-seg achieves a P of 97.0%, the highest among the compared models, with improved discriminative performance for sugarcane segmentation targets and a reduced false positive rate. Concurrently, the improved model achieved a R of 98.1%, surpassing all compared models. It exceeded the original YOLOv11n-seg by 1.8% and outperformed YOLOv5n-seg and YOLOv9t-seg by 2.4%. This demonstrates improved target sensitivity, thereby reducing false negatives. Regarding segmentation accuracy, Improved YOLOv11n-seg achieves mAP_0.5_ and mAP_0.5:0.95_ of 99.2% and 82.9%, respectively, outperforming all comparison models. Notably, mAP_0.5:0.95_ demonstrates a 1.8% improvement over the original YOLOv11n-seg, indicating improved robustness and localization accuracy for segmentation targets across different scales. Enhancing segmentation performance at higher IoU thresholds is of greater practical significance for accurate sugarcane impurity rate detection.

Regarding model parameter count and inference speed, Improved YOLOv11n-seg has 255,000 parameters, which is only marginally higher than that of YOLOv10n-seg. Compared with YOLOv8n-seg, YOLOv11n-seg, and YOLOv12n-seg, it achieves parameter reductions of 21.8%, 10.2%, and 9.3%, respectively. Its detection speed of 18 FPS is equivalent to YOLOv11n-seg, while substantially surpassing both YOLOv9t-seg and YOLOv12n-seg. The model thus balances real-time efficiency and detection accuracy.

**Table 2** presents the detection results for four sugarcane categories on the test set, including a comparison between the original and Improved YOLOv11n-seg models. As shown, Improved YOLOv11n-seg demonstrates varying degrees of improvement across all performance metrics for each category, indicating consistently enhanced detection performance. The enhanced model provides more precise segmentation boundaries. Among the categories, cane_top exhibits the most significant improvement, with R and AP_0.5:0.95_ improving by 2.8% and 3.5%, respectively. Furthermore, cane_segment and cane_top show relatively high performance metrics, whereas cane_leaves and cane_root show the lowest, primarily due to higher intra-class variability in color, shape, and texture.

**Table 2:** Test sets for each category before and after model improvement.

Class	YOLOv11n-seg				Improved YOLOv11n-seg			
	P	R	AP_0.5_	AP_0.5:0.95_	P	R	AP_0.5_	AP_0.5:0.95_
cane_segment	97.0	98.7	99.3	86.7	98.8	98.7	99.4	87.7
cane_top	96.2	97.4	98.7	87.9	97.8	98.5	99.4	88.6
cane_leaves	93.5	93.3	96.7	74.7	94.4	96.5	98.6	76.6
cane_root	95.8	95.8	98.7	75.2	97.0	98.6	99.4	78.7

In summary, a comprehensive analysis of multiple performance metrics indicates that Improved YOLOv11n-seg achieves a well-balanced trade-off between accuracy and efficiency, making it more suitable for sugarcane impurity segmentation tasks.

### 3.2 Ablation study results

To evaluate the effectiveness of the Improved YOLOv11n-seg model for sugarcane object detection, YOLOv11n-seg was incrementally enhanced with the C2_Ghost, C2_FSAS, ECA, and DySample modules. Model performance was evaluated through sequential module integration. The ablation test results for each configuration on the test set are summarized in **Table 3**.

**Table 3:** Ablation study.

Module				P	R	mAP_0.5_	mAP_0.5:0.95_	Parameters (×10^5^)	Speed (FPS)
C2_Ghost	C2_FSAS	ECA	DySample						
✓				95.6	96.3	98.3	80.8	**24.5**	**19.3**
	✓			96.3	97.4	98.9	82.5	29.3	17.4
		✓		97.4	95.7	98.8	81.8	28.4	17.9
			✓	96.7	97.4	98.8	82.0	28.5	18.2
✓		✓		96.4	96.4	98.8	81.4	24.5	19.2
	✓		✓	97.2	97.8	99.1	**82.9**	29.4	17.6
✓	✓	✓		**97.4**	96.6	98.9	82.5	25.4	18.6
✓		✓	✓	96.8	96.6	99.0	81.8	24.6	18.9
✓	✓	✓	✓	97.0	**98.1**	**99.2**	**82.9**	25.5	18.0

Bold text indicates optimal values.

The results indicate that progressively incorporating the C2_Ghost, C2_FSAS, ECA, and DySample modules consistently improves detection performance for sugarcane impurities, although the contribution of each module differs in magnitude. Overall, the Improved YOLOv11n-seg model incorporating all four modules attained the best overall performance in key metrics, namely P, R, mAP_0.5_, and mAP_0.5:0.95_.

Examining single-module contributions, the introduction of the C2_Ghost module reduced the model’s parameter count by 13.7% and increased detection speed by 7.2%, indicating its efficacy in reducing model complexity. However, incorporating C2_Ghost led to a slight reduction in mAP_0.5:0.95_, indicating a minor decrease in the model’s ability to accurately segment target regions. The incorporation of the ECA module increased P to a peak of 97.4%, highlighting the effectiveness of the channel attention mechanism in enhancing feature representation and suppressing false positives. Meanwhile, adding either the C2_FSAS or DySample module increased R, suggesting that these modules improve target coverage and reduce false negatives. Compared to the baseline model, the addition of any single module (except C2_Ghost) led to incremental improvements in mAP_0.5:0.95_, with C2_FSAS achieving the greatest gain of 1.4%, underscoring its crucial role in extracting semantic features for sugarcane objects. Regarding detection metrics, the model maintains high segmentation precision on the test set after integrating the C2_FSAS and ECA modules. This demonstrates that these modules do not induce overfitting under varying illumination conditions, indicating that the model exhibits strong generalization capability.

Among dual-module combinations, C2_FSAS+DySample delivered the most pronounced overall performance gains, with mAP_0.5_ and mAP_0.5:0.95_ values approaching those of the four-module configuration. The C2_Ghost+ECA pairing maintained a low parameter count while increasing inference speed to 19.2 FPS, outperforming YOLOv11n-seg across all other metrics. For the three-module combinations, the model achieved a more balanced performance; however, compared to the C2_FSAS+DySample combination, the R value decreased. After integrating all four modules, the Improved YOLOv11n-seg attained the highest R, mAP_0.5_, and mAP_0.5:0.95_ values of 98.1%, 99.2%, and 82.9%, respectively. Compared to models employing a single module, this indicates that multi-module fusion synergistically enhances feature extraction, contextual modelling, and boundary refinement, significantly improving the model’s segmentation capability for sugarcane objects.

In summary, ablation studies indicate that the enhanced model preserves high-precision object segmentation performance on the test set, reflecting robust generalization ability. Through the synergistic integration of its four modules, Improved YOLOv11n-seg achieves the highest overall performance in sugarcane impurity detection, substantiating the effectiveness of the proposed enhancement strategy.

### 3.3 Comparison of model segmentation results

**Figure 8:**
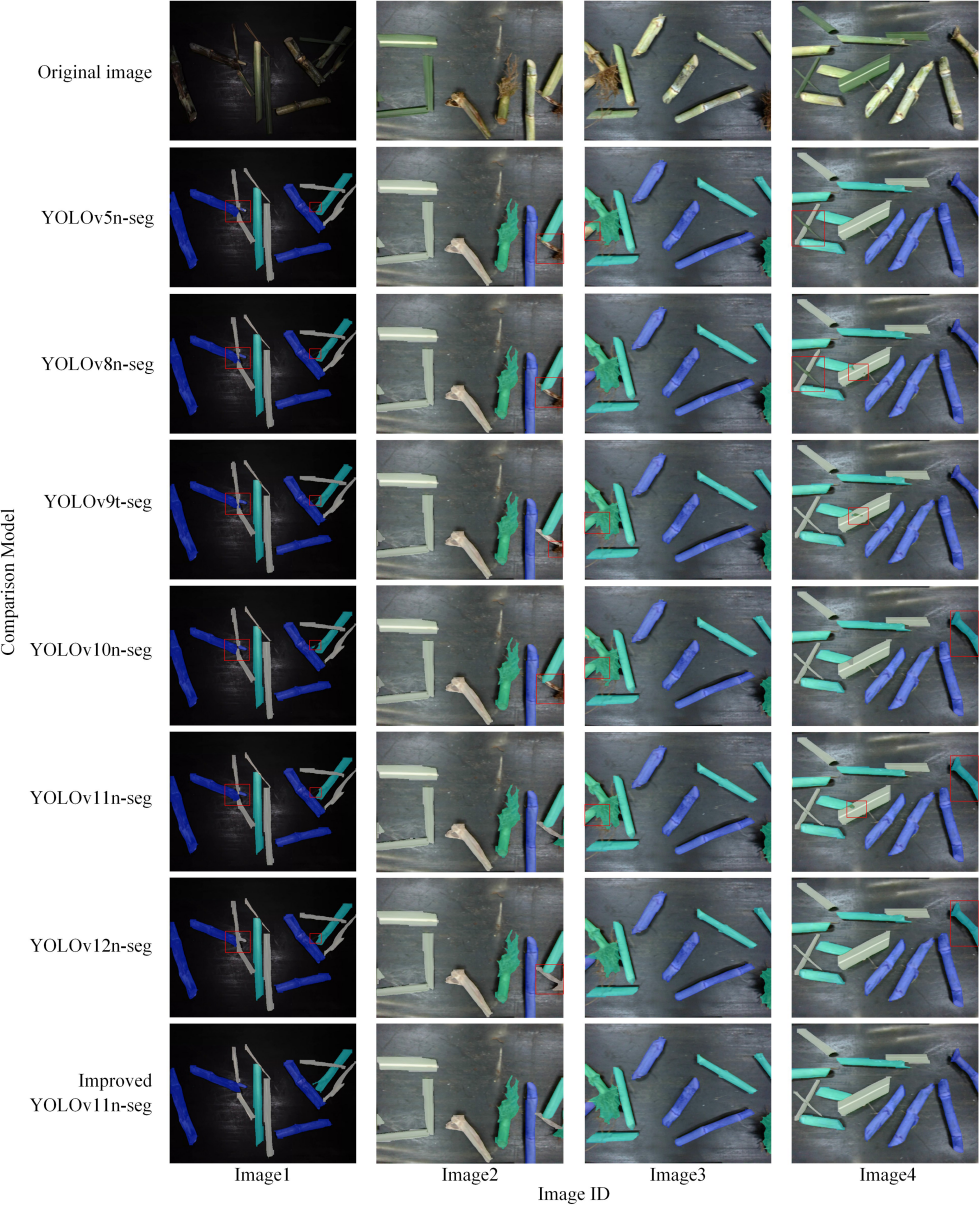
Detection results of different segmentation models. (Red rectangles indicate areas with significant segmentation discrepancies; each color mask corresponds to a distinct category.).

To validate the detection performance of our model against six other YOLO variants on sugarcane segmentation targets, each model was evaluated on the same set of sugarcane segmentation images. The segmentation results are shown in **Figure 8**. These results indicate that all seven models achieve satisfactory segmentation for the four target categories in most instances, with the Improved YOLOv11n-seg producing the most accurate and complete segmentation results. In Image1, Improved YOLOv11n-seg exhibited superior robustness in edge segmentation and category differentiation, whereas the other six models demonstrated incomplete segmentation and segmentation errors. YOLOv5n-seg, YOLOv8n-seg, YOLOv9t-seg, and YOLOv10n-seg all display missed detections, as illustrated in Image2. YOLOv9t-seg, YOLOv10n-seg, and YOLOv11n-seg generate false positives, as depicted in Image3. In Image4, YOLOv5n-seg, YOLOv8n-seg, YOLOv9t-seg, and YOLOv11n-seg exhibit missed detections, whereas YOLOv10n-seg, YOLOv11n-seg, and YOLOv12n-seg produce false detections. Overall, false negatives and false positives in the comparison models primarily occur when two segmentation targets are adjacent. When targets are isolated, feature extraction is more effective, resulting in accurate segmentation of most complete target regions. These results demonstrate that the proposed Improved YOLOv11n-seg outperforms the comparison models in edge segmentation precision and feature discrimination between categories, thereby demonstrating superior impurity recognition and regional segmentation accuracy.

The proposed model accurately separates sugarcane segments from various impurity categories, effectively reducing category confusion. It maintains precise segmentation boundaries even in transitional edge regions, making it highly suitable for sugarcane impurity detection tasks.

### 3.4 Feature output visualization

**Figure 9:**
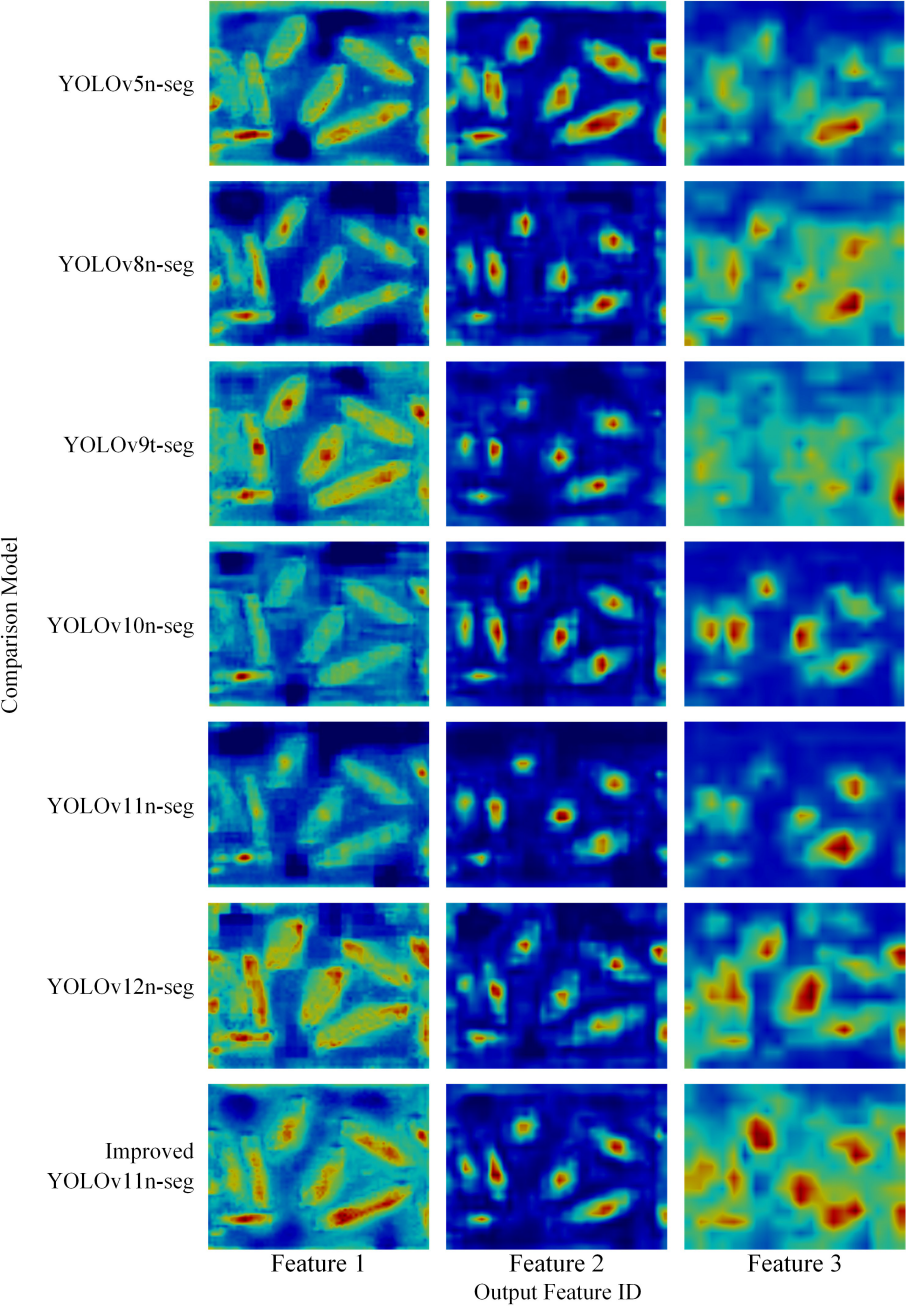
Visualization of feature outputs from different segmentation models. (Darker colors indicate higher levels of attention.).

To evaluate the feature extraction capability of the models, feature extraction visualizations were performed for Improved YOLOv11n-seg and six comparative models. The visualized feature maps correspond to the final outputs from the Neck module of each model. Utilizing Image3 from **Figure 8**, the results are shown in **Figure 9**. In Feature 1, the feature attention distribution across categories for YOLOv10n-seg and YOLOv11n-seg is significantly lower than in the other models. Conversely, Improved YOLOv11n-seg displays the highest overall feature attention, with more accurate spatial localization of attention regions. Its feature attention intensity gradually diminishes from the interior regions of the segmented object towards the peripheral regions. Compared to the comparative models, the feature distribution within individual segmented objects is more uniform in the proposed model. For instance, YOLOv5n-seg, YOLOv8n-seg, YOLOv9t-seg and YOLOv12n-seg exhibit the highest feature intensity at the center or ends of segmented objects, with lower intensity in other main body regions. Uniform feature distribution within a single segmented object aids the model in expressing the object’s overall semantic structure, thus enhancing segmentation boundary integrity. Conversely, features concentrated solely at central or localized areas may reduce the model’s capacity to precisely delineate the true object boundaries.

In Feature 2, the spatial distribution of features remains largely consistent, although their shapes differ significantly. For example, YOLOv11n-seg displays more circular features, whereas other models predominantly maintain strip-like features aligned with the object’s orientation. YOLOv5n-seg exhibits higher feature richness; however, its image edges contain redundant features that are not effectively filtered by the model. This can impede the accurate representation of category-specific features along image edges during subsequent feature extraction. In Feature 3, although YOLOv5n-seg demonstrates stronger feature expression in Feature 2 for segmentation targets, its overall feature representation in Feature 3 remains weaker than that of other comparative models. Furthermore, YOLOv8n-seg exhibits markedly lower attention to segmentation target features compared to Improved YOLOv11n-seg. The proposed model displays the strongest feature responses at the center of the segmented object, gradually diminishing towards non-segmented regions. Concurrently, non-segmented regions across different objects demonstrate specific inter-object feature correlations. As demonstrated by the detection results in Image3 for YOLOv8n-seg, YOLOv12n-seg, and Improved YOLOv11n-seg, this feature distribution not only enhances holistic perception of primary object regions but also facilitates discrimination between adjacent objects via inter-object feature correlations.

In summary, the visualization results in **Figure 9** further demonstrate that Improved YOLOv11n-seg achieves more accurate segmentation of edge information in sugarcane detection tasks and more effectively differentiates between segmented objects, thereby validating the contribution of each enhancement module to feature representation.

### 3.5 Model acceleration based on TensorRT

**Figure 10:**
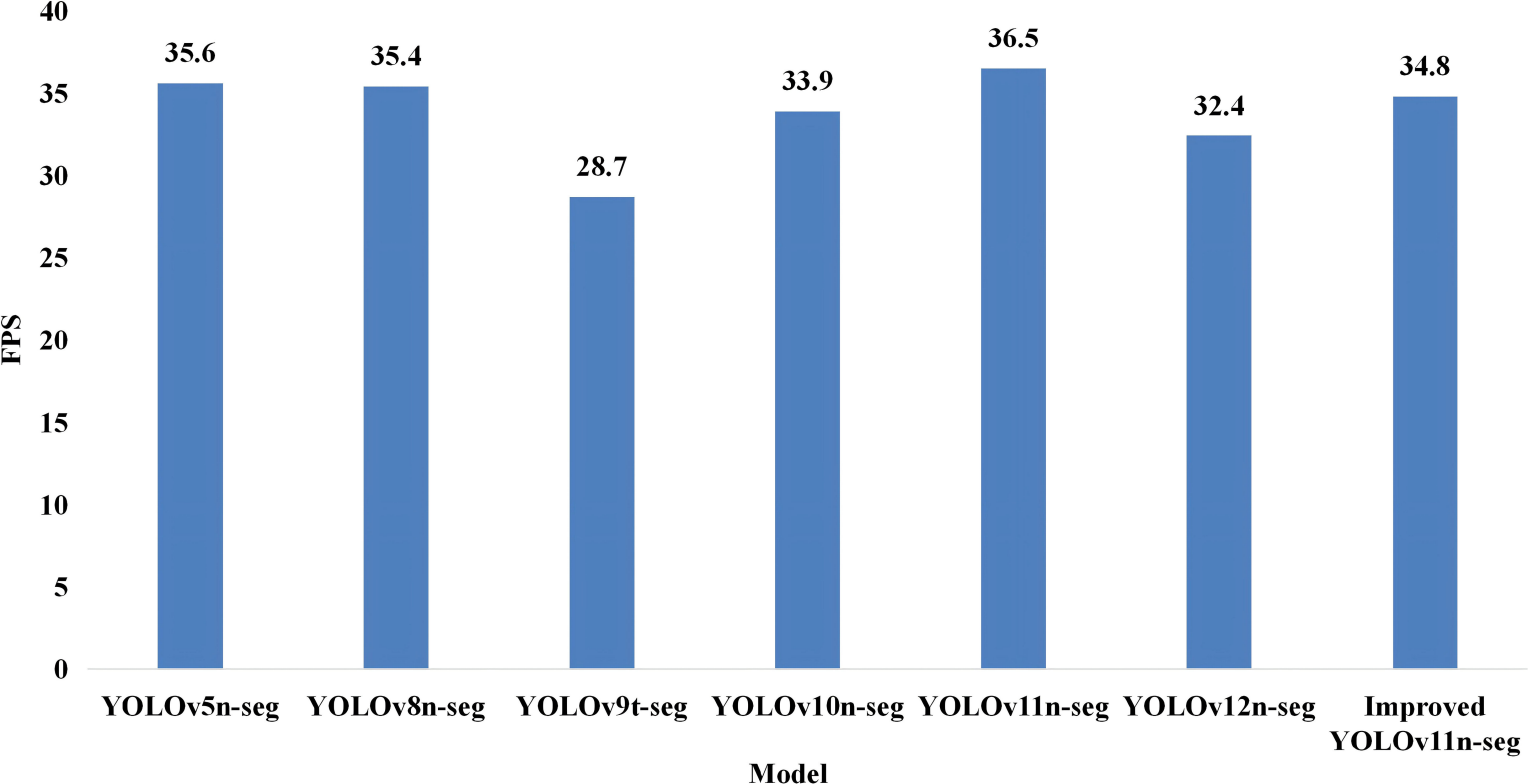
Comparison of model detection speed after TensorRT acceleration.

To further enhance real-time performance, half-precision (FP16) quantization using TensorRT was applied to reduce model size and accelerate inference. The detection speeds after acceleration for each model are presented in **Figure 10**. As shown in the figure, all models exhibit a substantial increase in inference speed after TensorRT-based FP16 quantization. Specifically, YOLOv11n-seg attained 36.5 FPS post-acceleration, suggesting that its architecture is particularly well-suited for accelerated execution on the Jetson Xavier NX platform relative to other models. Improved YOLOv11n-seg achieves 34.8 FPS after acceleration, corresponding to a 93.3% improvement in inference speed. Although its speed remains slightly lower than that of YOLOv11n-seg, it still outperforms all other comparison models. After TensorRT optimization, Improved YOLOv11n-seg consistently maintains a processing speed above 30 FPS, fully meeting real-time video-level detection requirements.

## 4 Discussion

In this study, edge devices are defined as embedded platforms featuring TensorRT acceleration. These platforms support FP16 and INT8 inference, thereby significantly reducing computational costs and memory usage while preserving detection accuracy and improving inference speed. This study proposes a lightweight segmentation model, Improved YOLOv11n-seg, for impurity detection in mechanically harvested sugarcane. Experimental results show that the model significantly improves segmentation accuracy while maintaining real-time performance on edge devices. The results validate the proposed enhancements: C2_Ghost reduces computational redundancy; C2_FSAS strengthens long-range frequency-domain feature modeling; ECA optimizes attention allocation among information channels; and DySample enables refined reconstruction of complex boundary details. Furthermore, the findings indicate that attention mechanisms and frequency-domain feature learning provide notable benefits in addressing challenges such as motion blur and texture similarity.

In practical sugarcane impurity rate monitoring, precise segmentation constitutes the foundation for accurate perception, whereas weight-based impurity detection necessitates establishing a mapping between visual features and weight. Subsequent research will investigate the relationships among area, geometric characteristics, and weight of different segmentation targets, facilitating the construction of a weight mapping model for each detection category. Converting segmentation outcomes into impurity weight estimates enables practical application within sugar mill processing workflows. Sensor installation during unloading, feeding, and secondary impurity removal stages enables continuous monitoring of sugarcane impurity rates. Detected impurity rates can provide guidance for raw material quality evaluation and pricing decisions. Alternatively, the system may be integrated into secondary impurity removal mechanisms to accurately detect and localize impurities, such as cane tops and roots, thereby facilitating precise removal. Moreover, deployment on sugarcane harvesters allows continuous estimation of impurity rates to optimize fan speed and conveyor parameters, enhancing impurity removal efficiency while minimizing crop loss.

To address a broader range of impurity types, future research will expand the dataset to incorporate inorganic impurities, thereby enabling more comprehensive sugarcane impurity detection.

## 5 Conclusion

1. The proposed Improved YOLOv11n-seg demonstrates superior overall performance in sugarcane impurity detection. Compared with the original YOLOv11n-seg, it achieves improvements in P, R, mAP_0.5_, and mAP_0.5:0.95_, reaching 97.0%, 98.1%, 99.2%, and 82.9%, respectively. This effectively reduces the risk of false positives and false negatives. With TensorRT acceleration, the model achieves a detection speed of 34.8 FPS on the Jetson Xavier NX, maintaining strong real-time performance. The enhanced model synergistically improves feature extraction and category discrimination through four modules, while reducing the total number of parameters by 10.2%. It combines high accuracy with a lightweight architecture, demonstrating the effectiveness of the proposed improvement strategy.
2. Ablation studies confirmed the efficacy of each enhancement module. Results indicate that the C2_Ghost, C2_FSAS, ECA, and DySample modules demonstrate complementary and synergistic contributions to feature representation. Specifically, C2_Ghost significantly contributes to reducing model parameters, C2_FSAS and DySample significantly improve mAP, while ECA substantially enhances precision. Thus, the collaborative design of multi-attention modules provides substantial improvements for sugarcane impurity detection tasks.
3. Segmentation results and feature visualization demonstrate that the proposed model exhibits enhanced representational capacity in semantic extraction and feature modelling. Compared to baseline models, it more effectively preserves segmentation boundary integrity and suppresses confusion between sugarcane segments and impurity categories. Overall, the proposed model combines high accuracy, lightweight architecture, and real-time performance, offering a reliable technical solution for detecting impurity rates in mechanically harvested sugarcane.

## Data Availability

The original contributions presented in the study are included in the article/supplementary material. Further inquiries can be directed to the corresponding authors.
